# Identification of potential drug targets and vaccine candidates in Clostridium botulinum using subtractive genomics approach

**DOI:** 10.6026/97320630015018

**Published:** 2019-01-31

**Authors:** Rati Sudha, Amit Katiyar, Poonam Katiyar, Harpreet Singh, Purushottam Prasad

**Affiliations:** 1 P.G.Department of Zoology,ANS College,Magadh University,Patna (Barh)-803213,India; 2 ICMR-AIIMS Computational GenomicsCentre,Division of I.S.R.M.,Indian Council of Medical Research,Ansari Nagar,New Delhi-110029,India; 3 NCC-Pharmacovigilance Program of India (PvPI),Indian Pharmacopoeia Commission (IPC),Raj Nagar,Ghaziabad-201002,India

**Keywords:** Botulism, drug target, vaccine target, subtractive genomics

## Abstract

A subtractive genomic approach has been utilized for the identification of potential drug targets and vaccine candidates in Clostridium botulinum, the causative agent of flaccid paralysis in humans. The emergence of drug-resistant pathogenic strains has become a significant global public health threat. Treatment with antitoxin can target the neurotoxin at the extracellular level, however, can't converse the paralysis caused by botulism. Therefore, identification of drug targets and vaccine candidates in C. botulinum would be crucial to overcome drug resistance to existing antibiotic therapy. A total of 1729 crucial proteins, including chokepoint, virulence, plasmid and resistance proteins were mined and used for subtractive channel of analysis. This analysis disclosed 15 potential targets, which were non-similar to human, gut micro flora, and anti-targets in the host. The cellular localization of 6 targets was observed in the cytoplasm and might be used as a drug target, whereas 9 targets were localized in extracellular and membrane bound proteins and can be used as vaccine candidates. Furthermore, 4 targets were observed to be homologous to more than 75 pathogens and hence are considered as broad-spectrum antibiotic targets. The identified drug and vaccine targets in this study would be useful in the design and discovery of novel therapeutic compounds against botulism.

## Background

Botulism is a rare disease occurring worldwide and is caused by botulinum toxin made by Clostridium botulinum bacteria. This disease is hard to control due to the emergence of drug-resistant pathogenic strains. Present treatment use antitoxin, which can target the neurotoxin at the extracellular level, however, can't converse the paralysis caused by botulism. To combat the current state, discovery of new drug targets is needed, which is known as the first step in the drug discovery process [1]. Traditional methods of drug target identifications are time consuming and overpriced, which cannot fight effectively against the rapidly growing multidrug-resistant bacteria. The success of human genome project as well as sequencing of many pathogenic bacteria has provided sufficient data that can be used to predict drug and vaccines targets using in-Silico approach [2, 3]. A subtractive genomics method along with Bioinformatics analysis is widely used approach for the drug target prediction [4]. This has open new routes for the identification of potential therapeutic candidates, which has accelerated the drug discovery process, maximizes the treatment alternatives and reduced the drug failure rate in the later phase of clinical trials saving lots of money. In the last decades, a subtractive genomics approach has been effectively used to predict drug targets in Pseudomonas aeruginosa [5], Mycobacterium tuberculosis [6], and Salmonella enterica [7]. The similar subtractive genomic approach has adopted in this study to predict potential drug and vaccine targets in C. botulinum, where pathogen-specific, essential proteins were mined from the analysis of chokepoint, pathway, plasmid, virulence and resistance proteins. Moreover, the subtraction dataset between the host and the pathogen genome offers a set of genes that are likely to be important to the pathogen but absent in the host. The identified pathogen-specific proteins have the potential to become a good target and might be used in the drug discovery process.

## Methodology

**Figure 1:**
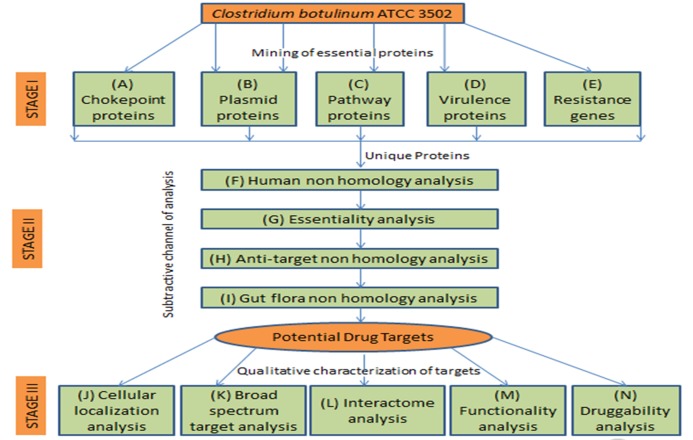
Flowchart describing the comprehensive methodology for the identification of potential targets, including drug and vaccine using subtractive genomics approach.

The probable drug targets in C. botulinum (strain Hall/ ATCC 3502/ NCTC 13319/ Type A) was identified through the subtractive genomics approach, including I) mining of essential proteins, II) subtractive analysis to exclude human homologs and III) qualitative characterization of probable targets essential for the growth of C. botulinum. A flowchart of the method used in this study is given in Figure 1.

## Stage I:

Mining of essential proteins The mining of essential proteins included the following analysis.

## Chokepoint proteins (CP):

Chokepoint proteins (enzymes) from C. botulinum and human host (labeled with A) were identified individually using Chokepoint Reaction Finder of BioCyc database [8]. The identical CP between human and C. botulinum was disqualified to avoid cross targeting and CP unique to C. botulinum (labeled with A1) was retained for further analysis. These proteins were further confined by their related enzymatic reactions (labeled with A2) in the KEGG database [9].

## Plasmid proteins:

Plasmid proteins of C. botulinum (labeled with B) were retrieved using NCBI [10].

## Pathway proteins:

Metabolic pathways in C. botulinum and human host (labeled with C) were obtained independently from the KEGG database [9] and subsequently compared to identify proteins present in distinct pathways of pathogens (labeled with C1) and shared pathways of pathogen and host (labeled with C2).

## Virulence proteins:

Virulence factors formerly reported from C. botulinum A str. ATCC 3502 (serotype A) was retrieved from the Virulence Factors Database (VFDB) [11] and scientific literature (labeled with D1). These proteins were manually examined for their existence in human pathways and proteins missing in the host were selected (labeled with D2).

## Resistance genes and protein network:

Orthologs of antibiotic resistance genes (such as gyrA, gyrB, rpoB, tetM and catD) in C. botulinum was obtained from the NCBI (labeled with E). The high confidence interactors (STRING score > 0.9) for resistance protein in C. botulinum were observed through STRING 10.0 [12] and the interactors absent in human pathways (labeled with E1) was selected for further analysis.

## Stage II: Subtractive channel of analysis

The collected proteins (labeled with A-E) were aligned with each other, and the projected unique proteins were passed through a series of subtractive analysis to predict possible drug targets in C. botulinum.

## Human non-homology analysis:

Proteins that are non-homologous to human (labeled with F) were filtered using BLASTp search against the non-redundant database of H. sapiens with E-value of 0.005.

## Essentiality analysis:

The identified non-homologous proteins were searched against the Database of Essential Genes (DEG) [13] with E-value of <0.0001 and the significant hits were retained (labeled with G).

## Anti-target non-homology analysis:

The identified essential proteins were filtered against the antitargets (essential proteins) of a host (n=210) with E-value of 0.005. The non-homolog proteins in the analysis were retained (labeled with H).

## Gut flora non-homology analysis:

The identified anti-targets were filtered against the proteins of human gut microbiota (n=66) using BLASTp with E-value of <0.0001 and sequence pairs with <50%. The shortlisted targets (labeled with I) were retained for qualitative characterization in stage III.

## Stage III:

 Qualitative characterization of the shortlisted targets The shortlisted targets (labeled with I) were characterized through cellular localization, broad spectrum, interactome, functionality, and druggability analysis.

## Cellular localization analysis:

Finding the protein location is significant to predict possible vaccine antigen candidates (surface proteins) and probable drug candidates (inner membrane and cytoplasmic proteins) in bacteria. The location of the shortlisted targets was predicted using Uniprot [14], CELLO [15], Gpos-mPLoc [16] and PSORTb [17].

## Broad-spectrum target analysis:

BLASTp search against the pathogenic strains (n=223) with an expected threshold value of 0.005 were performed to predict broadspectrum targets in C. botulinum. The shortlisted candidates were further mapped to Cluster of Orthologous Groups of proteins (COG) [18] resulting homologs in other pathogenic bacteria.

## Interactome analysis:

PPI network was constructed for the shortlisted targets using STRING 10.0 [12] and interactions with a STRING score > 0.9 were analyzed using Cytoscape 2.8.1 [32]. The targets (proteins) with a high degree or number of connections it has to other nodes were termed as 'hub' genes [19] and considered as probable drug candidates.

## Functionality analysis:

The function of the hypothetical proteins from the list of shortlisted targets was predicted using INTERPROSCAN [33].

## Druggability analysis:

Druggability of the shortlisted targets was analyzed using BLASTp search against the DrugBank 3.0 [20] with an e-value of 10^-5^. This is crucial to identify targets tested by FDA approved drugs. Targets non-homologous to the targets of the DrugBank were labeled as 'novel' targets.

## Results and discussion:

### Chokepoint protein (CP):

A total of 810 chokepoint reactions, including producing (325), consuming (305), and concurrently producing and consuming (180) from C. botulinum were generated (Table 1). The identical CP between human and C. botulinum was excluded which revealed 640 CP proteins unique to C. botulinum. It's an important step to avoid adverse health effects, as the possible drug may also target host's CP enzymes. Out of 640, enzymatic reactions of 295 were observed in the KEGG pathway. The protein catalyzing CP reaction is considered as a highly ranked metabolic drug target [21].

### Plasmid protein:

A total of 18 plasmid proteins from C. botulinum were retrieved using the NCBI databases (Table 1). Plasmids might play a key role in the adaptability of microbes by providing antibiotic resistance against a wide range of antibiotics and hence be a valuable drug target [22].

### Pathway protein:

A comparative metabolic pathway analysis of H. sapiens (280 pathways) and C. botulinum (92 pathways) revealed 23 distinct (present in C. botulinum and absent in H. sapiens) and 59 shared (present in both host and pathogen) pathways (Table 1). The associated proteins from distinct (325) and shared pathways (906) were further collected and the proteins common to C. botulinum and human host pathway were omitted to avoid cross targeting in this study. This approach includes the targeting of many specific highpriority pathways for the expansion of multi-drug resistance among pathogenic bacteria [23].

### Virulence proteins:

A total of 36 virulence proteins from Virulence Factors Database (9), literature (16), and proteolytic group I (11), associated with pathogenicity [24] were collected and excluding the repeats, 33 unique proteins absent in human pathways were selected (Table 1). Virulence factors expressed through bacteria are vital for the survival and growth of pathogenic bacteria and hence can be a valuable drug targets.

### Resistance genes and protein network:

Network analysis disclosed 107, 78, 33, and 30 associated interactors for orthologs of rpoB, tetM, gyrA, gyrB antibiotic resistance genes, respectively. Next, a total of 152 interactors absent in human pathways were selected which can be a good target (Table 1). In this study, no interactors were observed for catD resistance genes.

### Subtractive channel of analysis Human non-homologous:

Potential drug targets homologous to host could harmfully affect the host metabolism. Thus, avoiding proteins homologous to host is considered as the leading step in the identification of good drug targets [25]. In this study, qualified datasets from chokepoint protein, plasmid protein, pathway protein, virulence factors, and resistance gene analysis were subjected to non-homology analysis. A total of 1729 proteins were used for homology search and 893 non-homologous proteins were selected (Table 1).

### Essentiality:

The potential drug target must be a crucial protein for the survival of the pathogen [25].Out of 893 non-homologous proteins, 633 proteins were aligned significantly to Database of Essential Genes (DEG) and were considered as essential proteins for the survival of the pathogen (Table 1). The non-aligned 260 proteins were excluded from this analysis.

### Anti-target non-homologous:

Protein non-homologous to anti-targets (human essential proteins) is endorsed as drugs inhibiting such targets may interfere with host metabolism triggering severe toxic effects in the host [26]. In this analysis, 633 essential proteins were undergoing a homology search and homologous proteins with anti-target of the host were eliminated, whereas 483 non-homologous proteins were retained for further analysis (Table 1).

### Gut flora non-homologous:

The human host co-evolved with a gut microflora that plays an important role in the absorption of poorly digestible dietary components, vitamin synthesis, and degradation of xenobiotics [27, 28]. Gut microbiota also play a key role in human health by providing resistance to colonization of pathogens and opportunistic bacteria [28,29]. Therefore, deterioration of the gastrointestinal microbiota population may lack of first line of defense against invading bacteria and may also deal with the deficiency of nutrients in the host [28]. Anti-target non-homologous 483 proteins were undergoing a homology search and 18 gut flora non-homologous proteins were separated. Finally 15 potential targets were shortlisted after removing the redundancy (Table 1 and Table 2).

### Qualitative characterization of the potential drug targets:

Cellular localization:

In this study, we observed the cellular localization of proteins in cytoplasm (6 proteins), membrane (5 proteins), outer membranebounded periplasmic space (3 proteins) and extracellular (1 protein). Proteins that are located in the cytoplasm could be used as drug targets, whereas extracellular and membrane bound proteins are appropriate for vaccine targets [30].

### Broad spectrum:

Alignment of proposed targets with medically vital myco bacterial species and other bacterial pathogens facilitates the assessment of broad-spectrum antibiotics that attack a wide range of bacteria. The shortlisted targets were exposed to Cluster of Orthologous Groups of proteins (COG) hunt to identify homologs in other pathogenic bacteria and we observed four targets homologous to more than 75 pathogens and hence are considered as broad spectrum candidates.

### Interactome analysis:

A total 14 shortlisted targets were found to be interacting with different C. botulinum strains and protein CBO0881 has maximum 50 interactors. Target with higher interactors was considered as metabolically important active protein, and hence could be used as good drug target [31].

### Functionality analysis:

We did not observe any hypothetical proteins from the list of shortlisted targets and hence this step was excluded from this study.

### Druggability analysis:

The druggability of the proposed targets was evaluated by sequence similarity search against the targets from DrugBank [20] (Table 3). Drug target, CBO2715 is found to be homologous with DrugBank targets P26394, Q9HU21, O06330 and O52806 with their corresponding drugs DB02549, DB01694, DB04530 and DB01643, respectively. Remaining drug targets with no homology were differentiated as novel targets (Table 1 and Table 3), which require further experimental validation.

## Conclusion

In this study, we identified new targets, including drug and vaccine in C. botulinum using subtractive genomic approach. Identified targets were initially confirmed for their role in the survival of the C. botulinum. The downstream analysis includes non-similarity to gut flora proteins, and anti-targets in the host to predict highconfidence drug targets. Proposed targets were found to be an ideal broad-spectrum candidate, whereas interactome analysis highlighted the functional importance of many query targets. The adopted approach has the potential to be used as a general method for target identification and hence the proposed 15 targets might be used in drug discovery program.

## Figures and Tables

**Table 1:** : Drug target identification using subtractive genomics approach. The 15 potential targets including drug (6) and vaccine (9) candidates, non-similar to human, gut microflora, and anti-targets in the host were predicted in C. botulinum.

Drug target using subtractive genomics						
	Chokepoint (CP) [A]		Excluding repeats	Against Hsa (CP) [A1]		CP reaction in KEGG [A2]
Mining of essential proteins	810 Cbo proteins		726 Cbo proteins	640 Cbo proteins		295 Cbo proteins
(Stage-I)	Producer-325		Producer-300	Producer-294		Producer-131
	Consumer-305		Consumer-269	Consumer-194		Consumer-106
	Simultaneous-180		Simultaneous-157	Simultaneous-152		Simultaneous-158
	Plasmid protein [B]					
	18 proteins		18 proteins			
	Pathway protein [C]		Distinct pathways of Cbo against Hsa [C1]	Common pathways between Cbo and Has [C2]		
	92 Cbo pathways		Pathway- 23	Pathway- 59		
	280 Hsa Pathways		Proteins-325	Proteins-906		
	Virulence factors (VFD) [D1]			Unique proteins absent in human pathways [D2]		
	36 Cbo proteins			33 Cbo proteins		
	VFD- 9 proteins			VFD-9 proteins		
	VFD literature-16 proteins			VFD literature-16 proteins		
	VFD literature Group I (proteolytic)-11 proteins			VFD literature with Group I (proteolytic)-8 proteins		
	Resistance genes and protein network [E]			Unique interactors absent in human pathways [E1]		
	Resistance genes-5			Resistance genes-5		
	Interactors-253 proteins			Interactors-152 proteins		
Subtractive Channel of Analysis	Chokepoint protein		Plasmid protein	Pathway protein	Virulence protein	Resistance gene and proteins network
(Stage-II)						
						
Input proteins	295 proteins		18 proteins	1231 proteins	33 proteins	152 proteins
						
	Proteins passed-240		Proteins passed- 17	Proteins passed - 547	Proteins passed- 28	Proteins passed- 61
Human non-homology [F]	Excluded - 55		Excluded-1	Excluded- 684	Excluded-5	Excluded- 91
						
Essentiality [G]	Proteins passed-199		Proteins passed- 4	Proteins passed - 371	Proteins passed- 5	Proteins passed -54
	Excluded - 41		Excluded-13	Excluded - 176	Excluded - 23	Excluded - 7
						
Anti-target non-homology [H]	Proteins passed -100		Proteins passed- 4	Proteins passed- 320	Proteins passed- 5	Proteins passed- 54
	Excluded - 0		Excluded-0	Excluded- 2	Excluded- 0	Excluded- 0
	Repeats excluded-99			Repeats excluded -49		
Gut flora non-homology [I]	Proteins passed- 5		Proteins passed-3	Proteins passed-9	Proteins passed-0	Proteins passed-1
	Excluded - 94		Excluded- 1	Excluded- 311	Excluded- 5	Excluded- 52
						Cbo homolog failed subtractive analysis -1
Excluding repeats	Σ15 potential targets found					
Qualitative characterization of 15 targets (Stage-III)						
Cellular localization [J]		Broad spectrum target [K]		Interactome [L]	Functionality [M]	Druggability [N]
Cytoplasmic - 6		Alignment against the pathogenic strains:		Interactome-14 proteins	Hypothetical proteins were not observed hence excluded this analysis	Druggable - 1
Membrane - 5		Homolog >50 = 4		No Interactome-1 protein		Novel - 14
Outer membrane-bounded periplasmic space - 3		Homolog <50 = 11				
Extracellular - 1						

**Table 2:** Functional annotation of potential drug target and vaccine candidates in C. botulinum

No.	KEGG-ID	Associated pathway	Length (aa)	Description	Uniprot ID	CP Protein	*COG-ID
1	CBO0703	-	278	AraC family transcriptional regulator	A5HZP6	No	COG2207[K]
2	CBOP005	-	181	site-specific recombinase	A5I825	No	COG0582[LX]
3	CBOP15	-	430	putative bacteriocin biosynthesis protein	A5I835	No	COG0535[R]
4	CBOP17	-	391	TldD family protein	A5I837	No	COG0312[R]
5	CBO0311	cbo02010	342	iron compound ABC transporter permease	A5HYK4	No	COG0609[P]
6	CBO0385	cbo02010	290	phosphate ABC transporter permease	A5HYS9	No	COG0573[P]
7	CBO0962	cbo02010	260	molybdenum ABC transporter permease	A5I0F5	No	COG0555[P]
8	CBO1150	cbo02010	221	ABC transporter permease	A5I0Z1	No	COG0765[E]
9	CBO0945	-	179	Isochorismatase	A5I0D8	Yes	-
10	CBO0881	cbo02060	629	PTS system beta-glucoside-specific transporter subunit IIABC	A5I073	Yes	COG1264[G]
11	CBO2715	cbo00521, cbo00523	177	dTDP-4-dehydrorhamnose 3,5-epimerase	A5I5E9	Yes	COG1898[M]
12	CBO1432	cbo01503	283	N-acetylmuramoyl-L-alanine amidase	A5I1R2	Yes	COG0860[M]
13	CBO3111	cbo01503	967	N-acetylmuramoyl-L-alanine amidase	A5I6J2	Yes	COG0860[M]
14	CBO2695	cbo02020, cbo02040	272	Flagellin	A5I5C9	No	COG1344[N]
15	CBO3110	cbo01503	949	N-acetylmuramoyl-L-alanine amidase	A5I6J1	No	-

**Table 3:** Qualitative characterization of the potential drug target and vaccine candidates in C. botulinum. Druggability of the proposed targets disclosed 14 candidates as novel targets.

No.	KEGG-ID	Cellular localization	Broad spectrum (Number of pathogens homologs)	Interactors	Druggablility
1	CBO0703	Cytoplasmic	14	7	Novel
2	CBOP005	Cytoplasmic	94	2	Novel
3	CBOP15	Cytoplasmic	5	3	Novel
4	CBOP17	Cytoplasmic	78	1	Novel
5	CBO0311	Membrane	47	30	Novel
6	CBO0385	Membrane	4	12	Novel
7	CBO0962	Membrane	32	13	Novel
8	CBO1150	Membrane	49	10	Novel
9	CBO0945	Cytoplasmic	87	5	Novel
10	CBO0881	Membrane	24	105	Novel
11	CBO2715	Cytoplasmic	1	25	Druggable
12	CBO1432	**OMBPS	25	7	Novel
13	CBO3111	**OMBPS	25	7	Novel
14	CBO2695	Extracellular	85	23	Novel
15	CBO3110	**OMBPS	27	NIL	Novel
